# Image recognition-based petal arrangement estimation

**DOI:** 10.3389/fpls.2024.1334362

**Published:** 2024-04-04

**Authors:** Tomoya Nakatani, Yuzuko Utsumi, Koichi Fujimoto, Masakazu Iwamura, Koichi Kise

**Affiliations:** ^1^ Graduate School of Informatics, Osaka Metropolitan University, Sakai, Japan; ^2^ Graduate School of Integrated Sciences for Life, Hiroshima University, Higashi-Hiroshima, Japan

**Keywords:** plant measurement, tepal arrangement, segmentation, meta-learning, circular permutation matching

## Abstract

Flowers exhibit morphological diversity in the number and positional arrangement of their floral organs, such as petals. The petal arrangements of blooming flowers are represented by the overlap position relation between neighboring petals, an indicator of the floral developmental process; however, only specialists are capable of the petal arrangement identification. Therefore, we propose a method to support the estimation of the arrangement of the perianth organs, including petals and tepals, using image recognition techniques. The problem for realizing the method is that it is not possible to prepare a large number of image datasets: we cannot apply the latest machine learning based image processing methods, which require a large number of images. Therefore, we describe the tepal arrangement as a sequence of interior-exterior patterns of tepal overlap in the image, and estimate the tepal arrangement by matching the pattern with the known patterns. We also use methods that require less or no training data to implement the method: the fine-tuned YOLO v5 model for flower detection, GrubCut for flower segmentation, the Harris corner detector for tepal overlap detection, MAML-based interior-exterior estimation, and circular permutation matching for tepal arrangement estimation. Experimental results showed good accuracy when flower detection, segmentation, overlap location estimation, interior-exterior estimation, and circle permutation matching-based tepal arrangement estimation were evaluated independently. However, the accuracy decreased when they were integrated. Therefore, we developed a user interface for manual correction of the position of overlap estimation and interior-exterior pattern estimation, which ensures the quality of tepal arrangement estimation.

## 1 Introduction

Flowers are of highly diverse shapes in their body plants, for instance, in the number of perianth organs such as petals and sepals, and in the symmetry of the arrangements of the organs (Endress, 1999; Tucker, 1999; Smyth, 2018; Spencer and Kim, 2018). Among these characteristics, the arrangement symmetry changes from species to species, where close species may differ and distant species may not. Hence, the flower development process may transcend the difference in species. The formation of flower organs during flower development has been studied using mathematical models via computer simulation to understand the developmental bases of the flower shape diversity (Nakagawa et al., 2020). To verify such flower development models, comparing the positional arrangement of organs in blooming flowers with models is useful.

**Figure 1:**
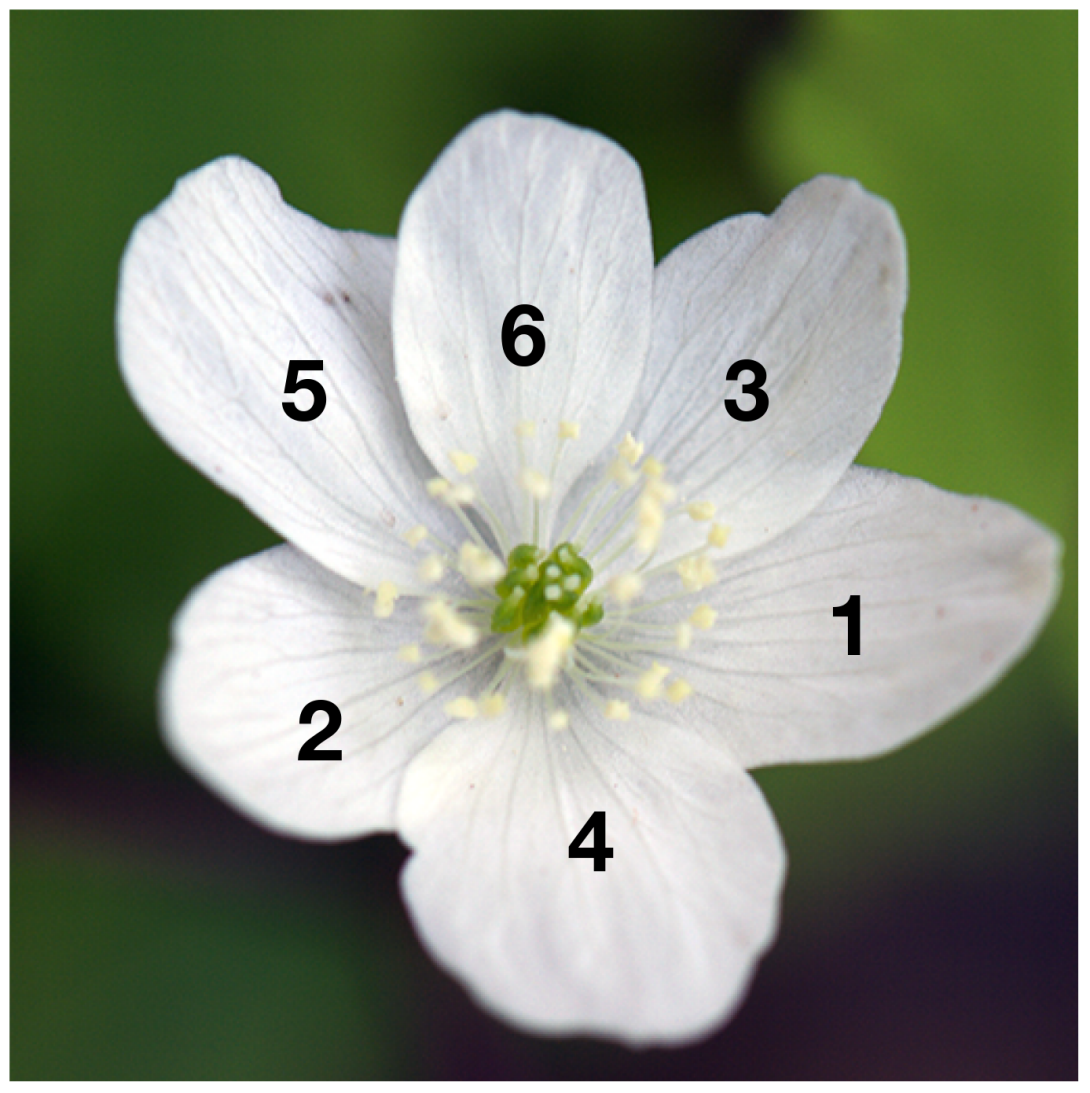
Example image of *Anemone* flower; index on each tepal (perianth organ) denotes the positioning, where exterior and interior organs take lower and higher indices, respectively.

This study aims to examine the positional arrangement of petal-like perianth organs (tepals). The development order of tepals can be estimated from the arrangement of neighboring tepals by studying a flower not fully bloomed (**Figure 1**). Exterior tepals are typically initiated earlier during the floral development in many clades of plant species, including *Anemone* and *Arabidopsis* (Smyth et al., 1990; Ren et al., 2010). At present, tepal arrangements are identified manually (Endress, 2010; Ronse De Craene, 2010; Vislobokov et al., 2014; Kitazawa and Fujimoto, 2018; Kitazawa and Fujimoto, 2020). The visual examination of a large number of flower images is extremely labor intensive; moreover, judging these images unless one is a specialist is difficult. Thus, this study aims to automate this task to reduce the burden on researchers.

**Figure 2:**
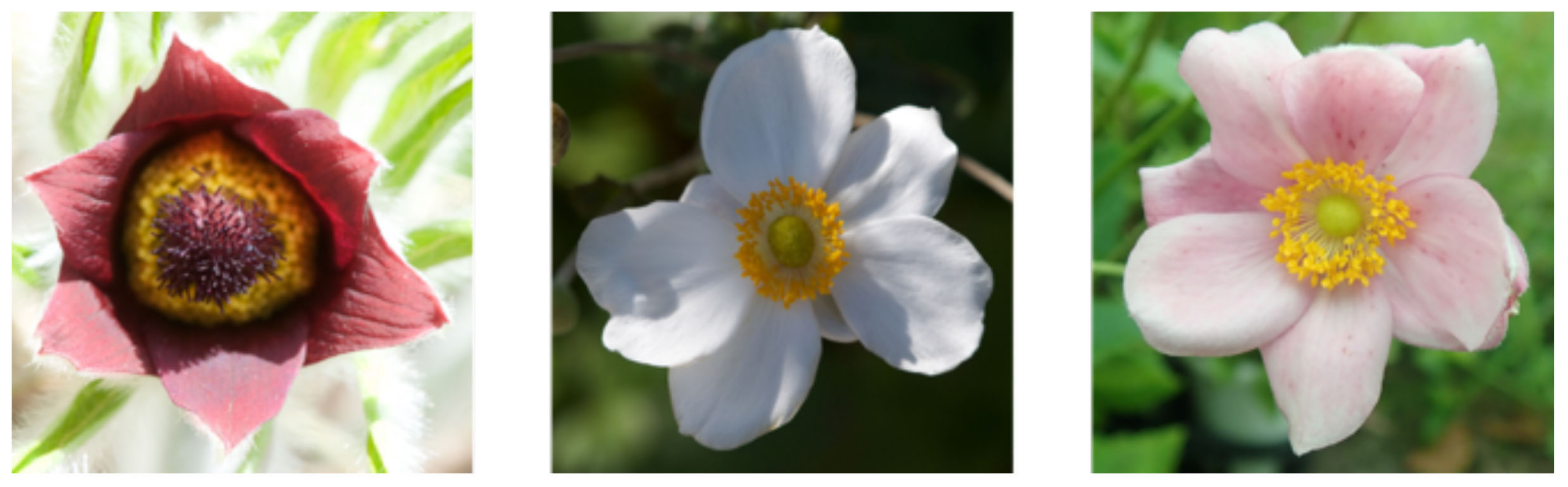
Floral shape and color diversity in *Anemone* species, *A. pulsatilla* (left) and *A. x hybrida* (center, right).

We use image data for several *Anemone* species and cultivars that were collected for the development of the flower shapes model. Every image in this data was manually captured when the flowers were blooming and shows how the tepals are arranged. For this reason, the number of flower images that can be used for machine learning is small, unlike large-scale datasets such as ImageNet (Deng et al., 2009) or Microsoft Common Objects in Context (MS COCO) (Lin et al., 2014) that are often used in image recognition. In addition to the small number of images included in these data, the lack of diversity in the shapes of the tepals is a problem when these images are used. The appearance of the tepals is quite different for individual flowers (diversity), and due to the small number of pictures (small quantity), there are not many images showing flowers that have a similar appearance (**Figure 2**). Therefore, it is difficult to build an end-to-end classifier that takes images as input and then outputs tepal arrangement.

Therefore, we propose estimating the tepal arrangement from the interior-exterior pattern of the overlap between tepals. This method detects the overlapping parts of the tepals, extracts the interior-exterior patterns of the tepals, and then identifies the tepal arrangements through circular permutation matching. Considering that there is only a small amount of training data, flower detection is performed using the You Only Look Once (YOLO)^1^ algorithm, which is capable of object detection after fine-tuning on a small number of training images. Moreover, the recognition of flower regions and the detection of overlapping parts are conducted by segmentation using GrabCut (Rother et al., 2004), which does not require training, and corner detection using the Harris corner detector (Harris and Stephens, 1988). Additionally, meta-learning, which is a machine learning framework that enables effective learning using a small amount of data, is used for the identification of interior-exterior relationships of the tepals.

Circular permutation matching, which is used to estimate the tepal arrangement, cannot estimate correctly if even one result for the overlapping part or the interior-exterior relationship is incorrect. Also, due to the property of pattern recognition, the detection of overlapping parts and the recognition of the interior-exterior relationship are not perfect. Thus, the accuracy of the estimation of tepal arrangement decreases due to these errors. Therefore, we attempt to improve the estimation accuracy using manual correction of the detection and recognition results. The result of the study showed that with total automation, the estimation accuracy of tepal arrangement was 0.275. However, after manual correction, the estimation accuracy increased to 0.711.

## 2 Related work

Here, we introduce studies in which image processing was used for flower image observation in plant measurement and studies related to tepal overlapping order estimation. As a flower is a familiar object, it is one of the commonly used image subjects in the fields of computer vision and pattern recognition. ImageNet (Deng et al., 2009), which is a representative common-object image dataset, also contains a category for flowers. Moreover, species recognition by flower appearance has been studied because flowers have diverse shapes and colors. Oxford flower datasets (Nilsback and Zisserman, 2006, 2008), are well-known and widely used in the field of computer vision. In this datasets, there are two categories; one comprises 17 types of images, where each type contains 80 images; the other comprises 102 types of images, where each type contains 40 to 258 images. Many image recognition studies have used the Oxford flower datasets (Nilsback and Zisserman, 2006, 2008; Fernando et al., 2012a, b, 2014; Hu et al., 2014; Mabrouk et al., 2014; Xie et al., 2014; Yang et al., 2014; Wang et al., 2020). Additionally, segmentation has also been carried out using the datasets (Nilsback and Zisserman, 2010; Chai et al., 2011; Chai et al., 2012). Moreover, a method that performs recognition through the production of a unique dataset (Guru et al., 2011) has been proposed, as has an interactive segmentation method (Zou and Nagy, 2004). There are studies on flower recognition that do not analyze flower structure.

In the field of agriculture, flower detection in images is being studied for the automation of farm work and the prediction of crop yield. In indoor environments such as greenhouses, flower detection methods for the automation of tomato flower picking (Rahim and Mineno, 2020) and the automatic pollination of raspberries and blackberries (Ohi et al., 2018) have been studied. Furthermore, flower detection in crops grown outdoors is being done with deep learning, which offers object detection that is robust in any lighting conditions. Flower detection from images is currently in use for crop prediction and the automation of tasks such as picking flowers and pollinating strawberries (Chen et al., 2019; Lin et al., 2020), apples (Dias et al., 2018; Wu et al., 2020), grapes (Millan et al., 2017; Liu et al., 2018), and kiwis (Williams et al., 2020). However, these are methods for flower location detection that do not analyze flower structure.

Some studies modeled the shape details of flower organs such as petals. A method that reproduces the detailed shapes of flowers, including their petals, from computed tomography images (Ijiri et al., 2014), methods that chronologically model the flower blooming process (Ijiri et al., 2008; Yan et al., 2014), and a method that reconstruct shapes of flowers in actual images using the RGB-D image data and petal model of color and shape that was prepared in advance (Zhang et al., 2014) have been developed. These models were built to draw flowers using computer graphics, and they require the measurement data for three-dimensional (3D) shapes and the application of 3D models. Thus, they are not suitable for analyzing the shapes of flowers that bloom outdoors.

## 3 Materials and methods

### 3.1 Dataset

In this study, we used 3,942 flower images of several *Anemone* species: *A. flaccida, A. hepatica*, *A. x hybrida, A. nikoensis, A. pulsatilla, and A. soyensis*. These images were photographed in locations with Shiga, Kyoto, Hyogo, Okayama, and Hiroshima Prefecture, using an Olympus TG-5 digital camera and a Nikon D5200 SLR camera. The image resolutions were 568 × 3,712, 4,928 × 3,264, 4,000 × 3,000, and 4,000 × 2,672 pixels; all images were saved in JPEG format. The size of the images was normalized to 1,000 × 662 pixels for the experiment. The images used in this study can be found in the data repository: 10.6084/m9.figshare.25323112.

Bounding boxes to indicate the position of the flowers were attached to all of these images. Moreover, the position of tepal overlap in the bounding boxes, and interior-exterior relationship of the tepals were studied and manually labeled. As the tepal arrangement of each pair of neighboring tepals is uniquely determined by their interior-exterior pattern, the arrangement of tepals was correctly labeled according to the interior-exterior relationship of the overlapping tepals. Moreover, labels to indicate the flower region at the pixel level were manually attached inside the bounding boxes in 420 images.

### 3.2 Tepal arrangement

**Figure 3:**
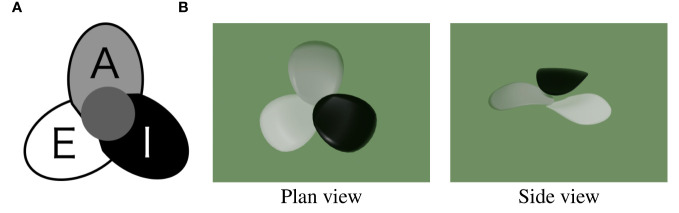
Class label I, E and A indicating the interior-exterior relations of tepals. **(A)** 2D diagram **(B)** 3D visualization^^2^^.

Tepal arrangement represents the number of tepals and their interior-exterior relationship; it is determined by the tepal development order. The interior-exterior relations of tepals can be represented as a circular permutation by attaching a class label to each tepal. There are three types of class labels (**Figure 3**): I (Internal), where a tepal is above both the tepals on its sides; E (External), where a tepal is below both the tepals on its sides; and A (Alternating), where a tepal is above the neighboring tepal on one side and below on the other. The tepal arrangements represented as circular permutations that match with each other after rotation or reflection transformation are treated as the same arrangement. Only some of all the conceivable arrangements occur in nature, and one-three types have been identified for each tepal number.

**Figure 4:**
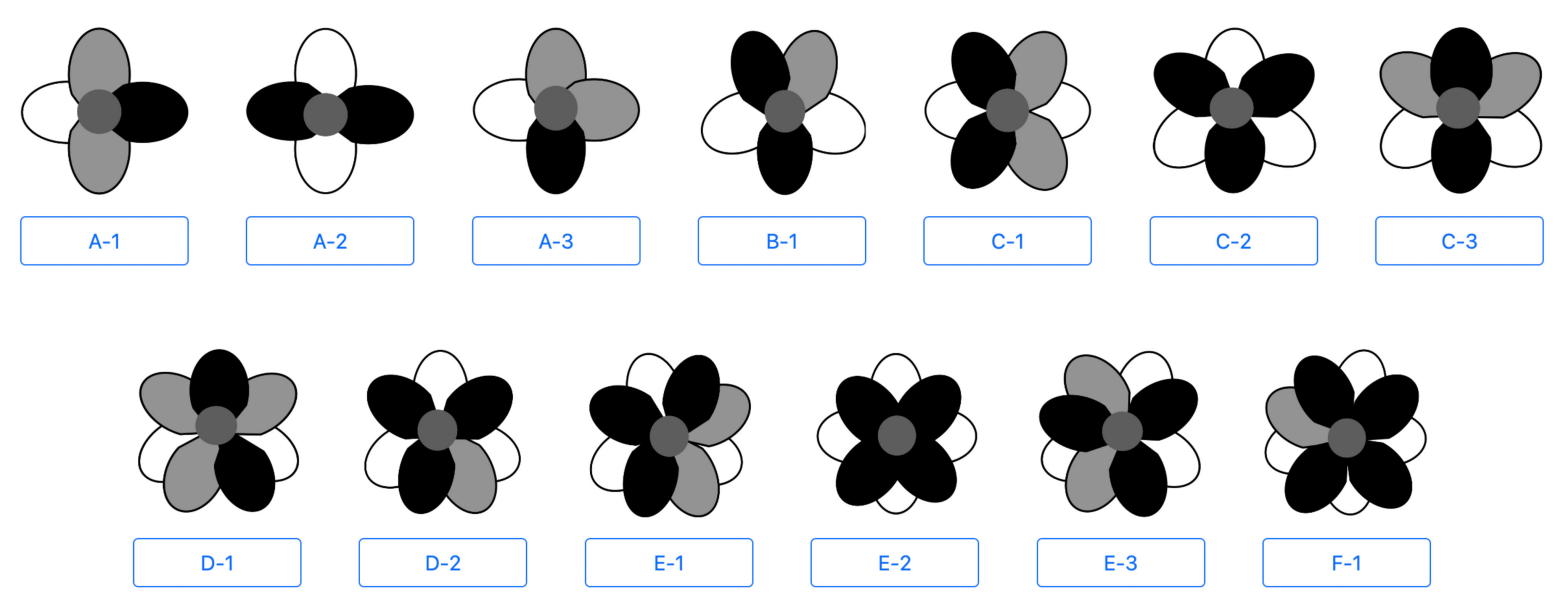
Diagram of tepal arrangement model.

In this study, *Anemone* species and cultivars having 4–9 tepals are studied. **Figure 4** shows a diagram of a model that includes 13 of the tepal arrangement types frequently observed in flowers with these tepal counts. The color of each tepal in this diagram indicates its class label; black, gray, and white represent I, A, and E, respectively. Moreover, the letter shown in the lower part of the diagram indicates the number of tepals (A–F correspond to 4–9, respectively), and the number indicates the tepal arrangement order for each tepal number.

### 3.3 Estimation of tepal arrangement

**Figure 5:**
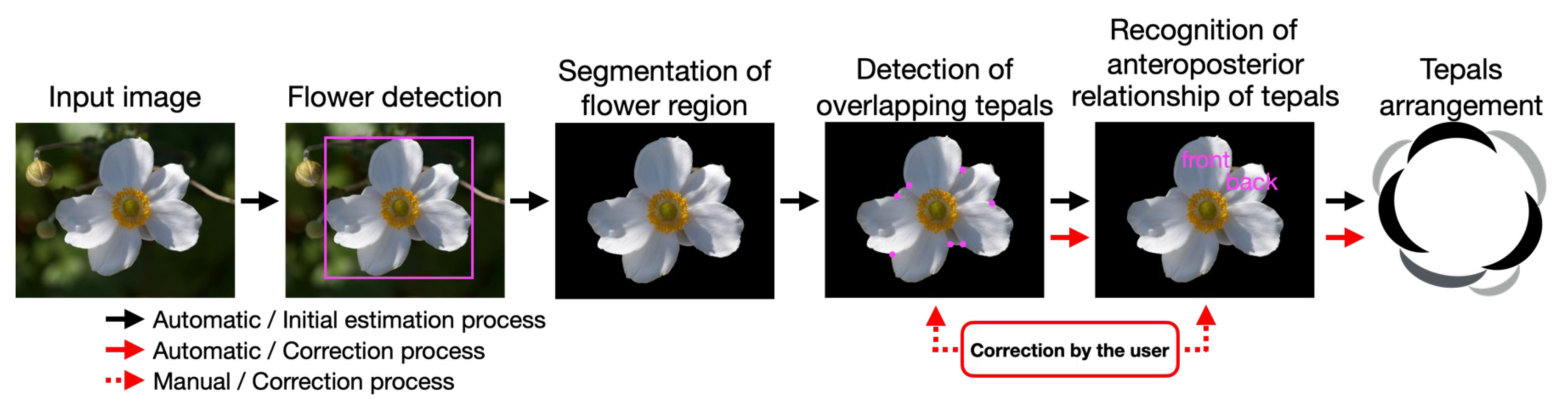
Overview of proposed method.

**Figure 5** shows the process flow for the proposed method. Tepal arrangement, which is the object to be estimated, can be uniquely determined from the interior-exterior relations between all of the overlapping tepals. Thus, the overlapping parts of the tepals and their interior-exterior relations are estimated after the flower region is obtained through flower detection and segmentation. Finally, the tepal arrangement is estimated using the estimated interior-exterior relations. In the following section, we describe the details of flower detection, segmentation, tepal overlap detection, the estimation of interior-exterior relations, and the tepal arrangement estimation.

#### 3.3.1 Flower detection

For the preprocessing, the proposed method carried out flower detection in the original image to obtain a rectangle that indicated the position of the flower (the bounding box). The proposed method used YOLO ver. 5 ^3^ for flower detection. YOLO v5 has shown good accuracy in common-object detection, and it is expected to be highly accurate in flower detection after fine-tuning, even if the training dataset is small. YOLO v5 simultaneously detects and classifies objects using a convolutional neural network, and the original model could detect and classify 80 objects. As the proposed method only needs to detect the flower region, we fine-tune the model using flower images.

#### 3.3.2 Flower region segmentation

Because the estimation of the tepal interior-exterior relationship is affected by the image background, the flower region is extracted from the detected image. As the flowers have a color that is different from their background, the proposed method utilizes GrabCut (Rother et al., 2004), which is a color-based segmentation technique, to segment the flower region. GrabCut employs a Gaussian mixture model to represent the color distribution of the foreground and background, and it performs the segmentation using this model. Because the model is estimated individually for each image, no prior training is required. The color distribution model is constructed based on a bounding box that encompasses the user-defined foreground region. As the bounding box defined in 3.3.1 encompasses the flower region, we use this bounding box to automatically construct the model and segment the flower region instead of using the user-specified rectangle.

#### 3.3.3 Overlapping tepal detection

**Figure 6:**
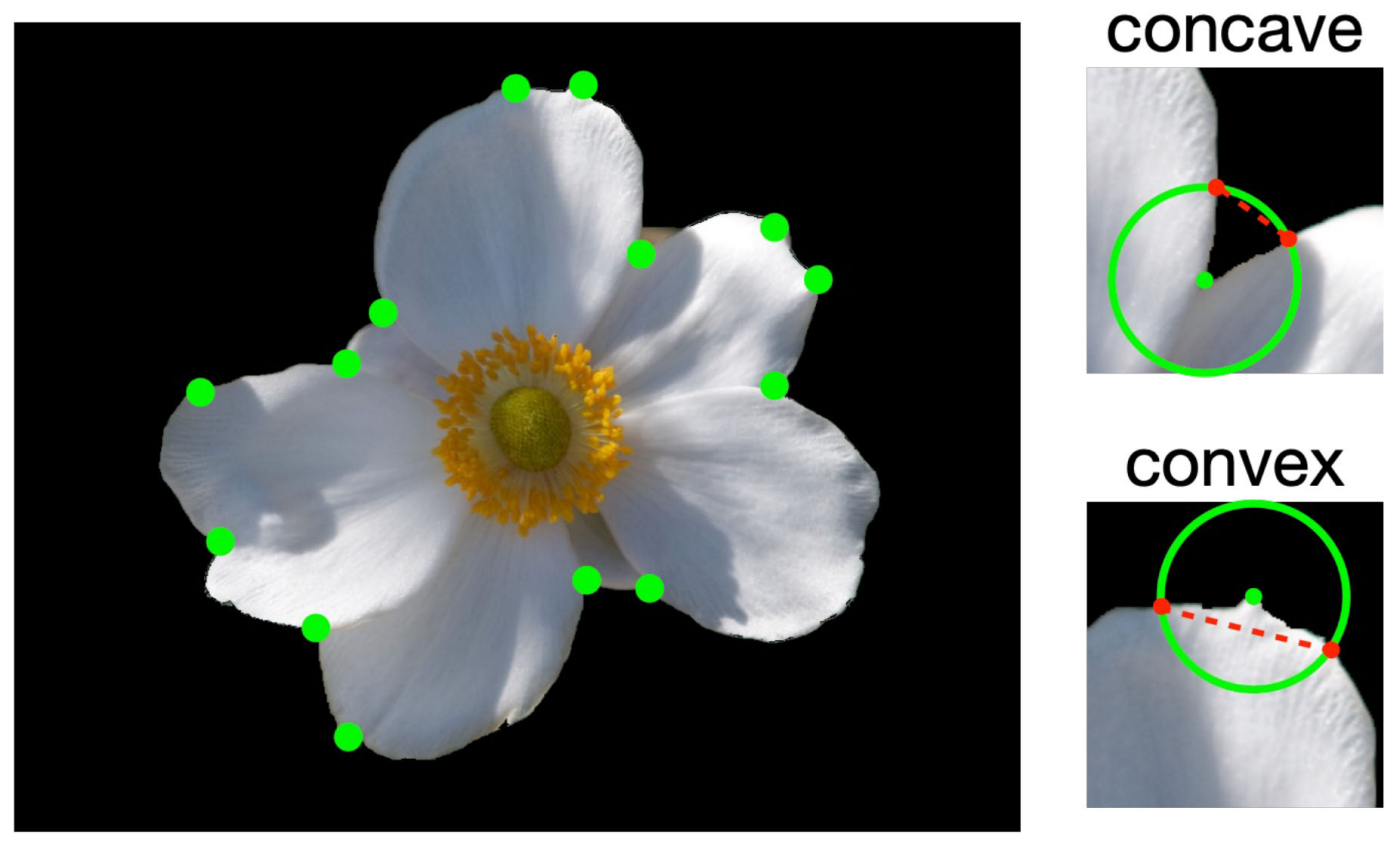
Detected corners (green points) and convex/concave corners in surrounding boundaries.

Next, the points at which the tepals overlap are detected. The points are detected by using the Harris corner detector (Harris and Stephens, 1988). As the corner detector is not perfect, the detected points contain false positives (**Figure 6**, left image). This image shows that when the region around a detected point is examined, the overlapping point is found to be concave, whereas false positives are convex, as shown in the image on the right side of **Figure 6**. Therefore, the proposed method detects the overlapping points by using this convex/concave characteristic as follows:

1. Draw a circle around the detected point, as shown in green in the close-up image of a corner on the right side of **Figure 6**, and obtain two points that intersect with the contour of the tepal (red points in the figure).
2. Draw a straight line that connects the two obtained points (red broken line, **Figure 6**). If the entire line is above the foreground, the point is considered to be a false positive and is removed.
3. When the entire line is above the background, it is determined to be an overlapping point.

#### 3.3.4 Recognition of interior-exterior relationship between tepals

**Figure 7:**
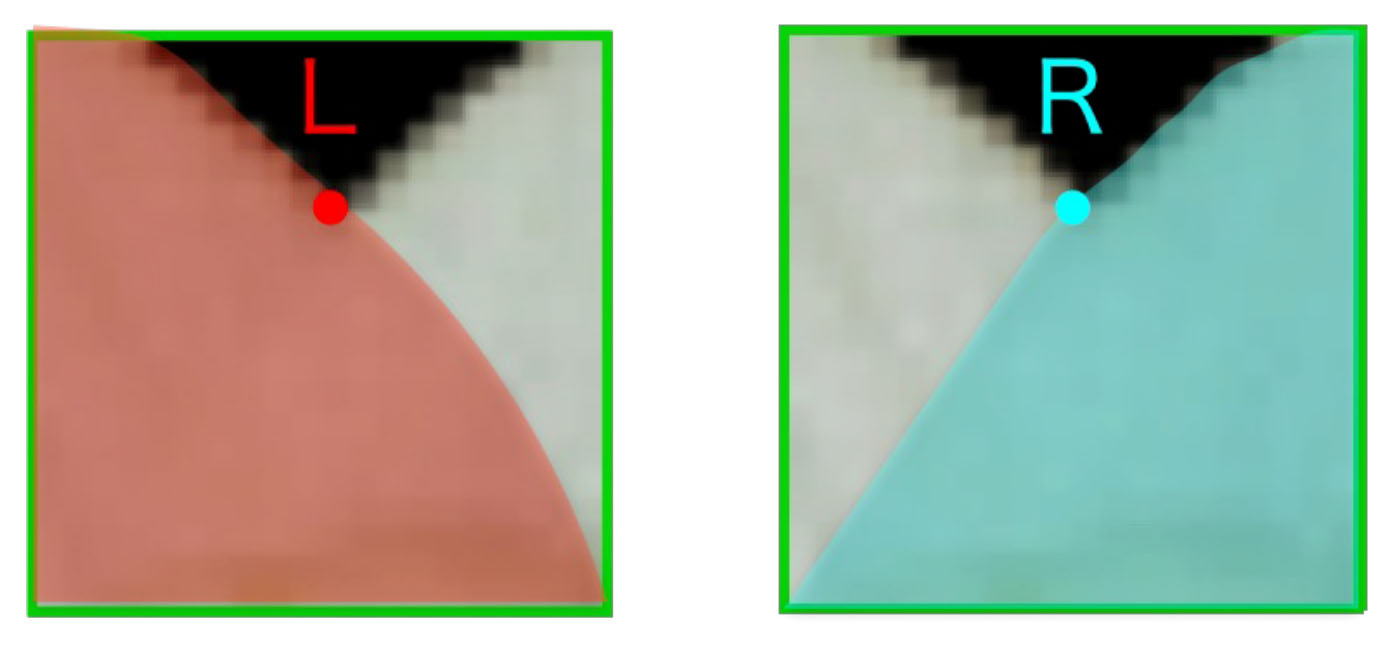
Examples from left-(L) (left) and right-tepal-forward class (R) (right).

The recognition of the interior-exterior relation of tepals is solved as a binary classification problem for images of tepal overlapping regions. The two classes are the left-tepal-forward class (L) and the right-tepal-forward class (R) (**Figure 7**). The images of tepal overlapping regions (the patches) are cropped from the segmented images around the overlapping points.

Each flower in the images used in this study has tepals of a different color and shape from the other flowers. Therefore, the appearance of the cropped patches is diverse. Moreover, the number of patches that can be cropped from one segmented image is 6 or 7 at most. This number of patches is insufficient for training a classification model; therefore, the model cannot achieve sufficient classification accuracy. Thus, this study employs synthesized petal overlapping patches (hereafter referred to as synthetic patches) and meta-learning to achieve sufficient accuracy. Synthetic patches are used to compensate for the small number of cropped patches and to generate an adequate number of patches for training. Meta-learning is used to prevent a reduction in classification accuracy due to differences in the appearance of the flowers.

##### 3.3.4.1 Generate synthetic patches

**Figure 8:**
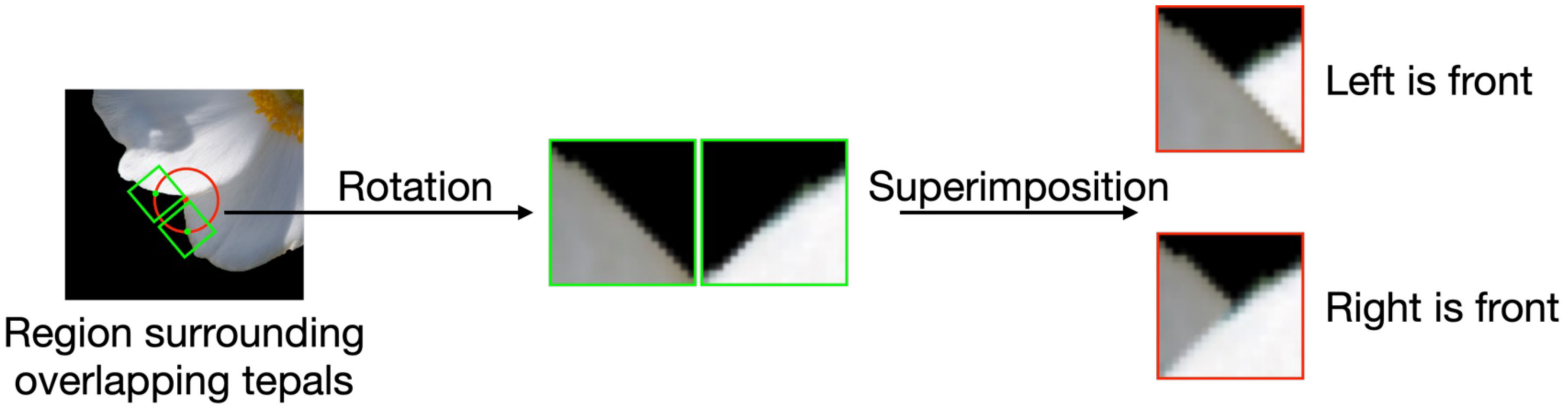
Procedure for generating synthetic patches.

**Figure 8** shows how the synthetic patches are generated through image processing. First, we draw a circle with the center as the position of overlap of the tepals, as shown in red on the left side of **Figure 8**. Next, we crop two patches, which are shown as the green squares centered at the intersections of the red circle and the contour on the left side of **Figure 8**. These patches are called the original patches. The radius of the red circle must be set so that the cropped region does not contain the overlapping parts of the tepals. Lastly, by superimposing these two patches, a synthetic patch that simulates the overlapping of the tepals is generated. As it is obvious whether the synthetic patches belong to L or R classes, the correct class label can automatically be applied to the synthetic patches; this cannot be done for the cropped patches.

##### 3.3.4.2 Meta-learning

Next, model-agnostic meta-learning (MAML) (Finn et al., 2017), which is one of the meta-learning methods, is used to classify the cropped patches. Meta-learning is a framework that optimizes the learning of new tasks through learning various tasks. The object of optimization is different for each method. In MAML (Finn et al., 2017), the optimization is done by learning the initial model of a network that is capable of accommodating many different tasks.

**Figure 9:**
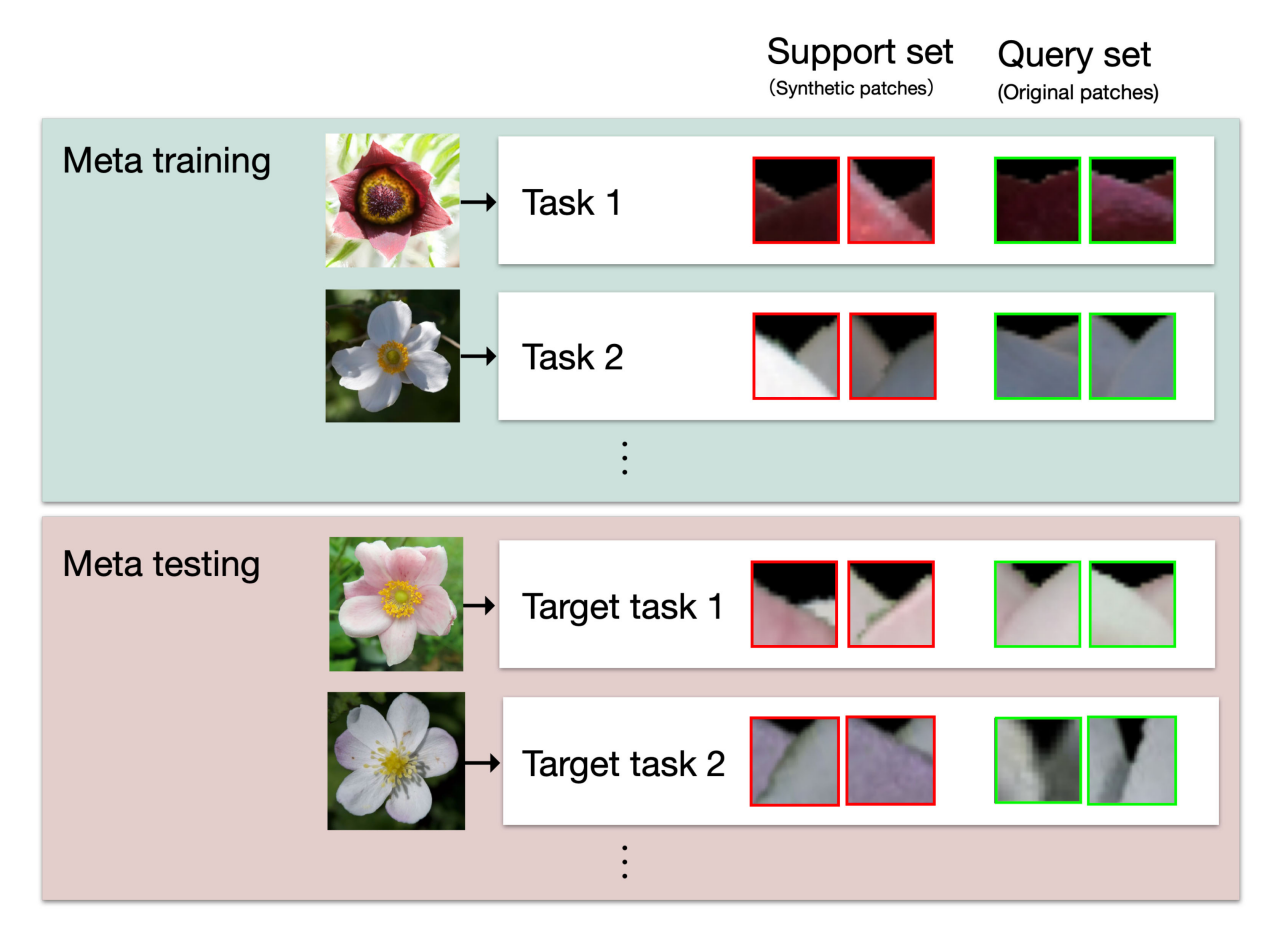
The meta-learning framework for this study. Each task consists of patches generated or cropped from a single flower. Support sets, which are used to train or optimize the model parameters, consist of synthetic patches. Query sets, which are used to evaluate the model, consist of original patches. In the training process, meta-training tasks are used to train and evaluate the model. In the evaluation process, meta-testing tasks are used for model optimization and evaluation.

**Figure 9** shows the meta-learning framework used in this study. The tasks used for learning the model are called the meta-training, and the target tasks are called the meta-testing. Each task consists of a support set and a test set. The support set includes the training data, which are used for training the model, and the query set contains the test data for evaluating the model. In MAML, the model is trained on the support set within a meta-training task to derive parameters that are appropriate for the task. Next, the performance of the model is evaluated using a query set in the meta-training task and the model parameters are updated based on the error of the test data. By iterating this process multiple times for each task in the meta-training, the model is trained to rapidly adapt to the various tasks. Finally, the model is evaluated using meta-testing. The model parameters that were trained by meta-training are updated using the support set for the meta-testing, and then the performance of the updated model is evaluated using the query set for the meta-testing.

In this study, each task consists of patches generated from a single flower, and synthetic patches are used for the support set, while the query set is the original patches for the interior-exterior estimation, as shown in **Figure 9**. In the training process, the model is trained using synthetic patches from a support set of a meta-training task and evaluated using original patches from a query set of a meta-training task. The process is iterated for the number of meta-training tasks. Then, in the estimation process, the parameters of the model are optimized for a meta-testing task using the support set of the task, and the estimation is performed on the query set of the task with the optimized parameters.

#### 3.3.5 Tepal arrangement estimation

The tepal arrangement estimation is performed using the arrangement from the interior-exterior relation recognition results. The estimation consists of three steps.

**Figure 10:**
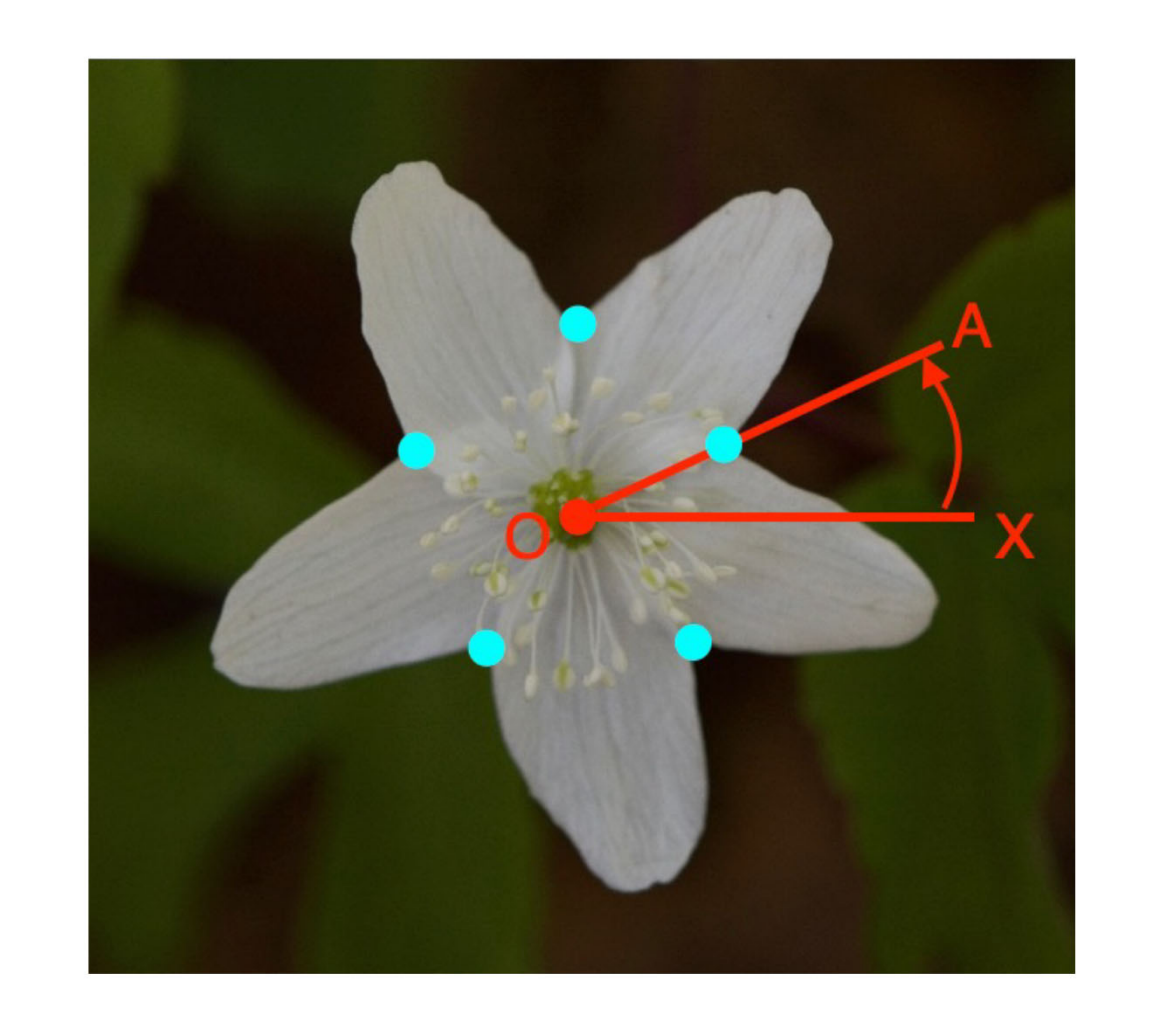
Acquisition of a circular permutation of overlapping tepal classes.

First, we obtain the class labels (L, R) of the tepal overlapping as a circular permutation. We set the center of gravity as the origin O (**Figure 10**) and consider the angle between the red line, OX, in the horizontal rightward direction from the origin and the line connecting the overlapping points (light blue) and the origin. The circle permutation is obtained by arranging the class labels in the order of increasing angle at each overlapping point.

**Figure 11:**
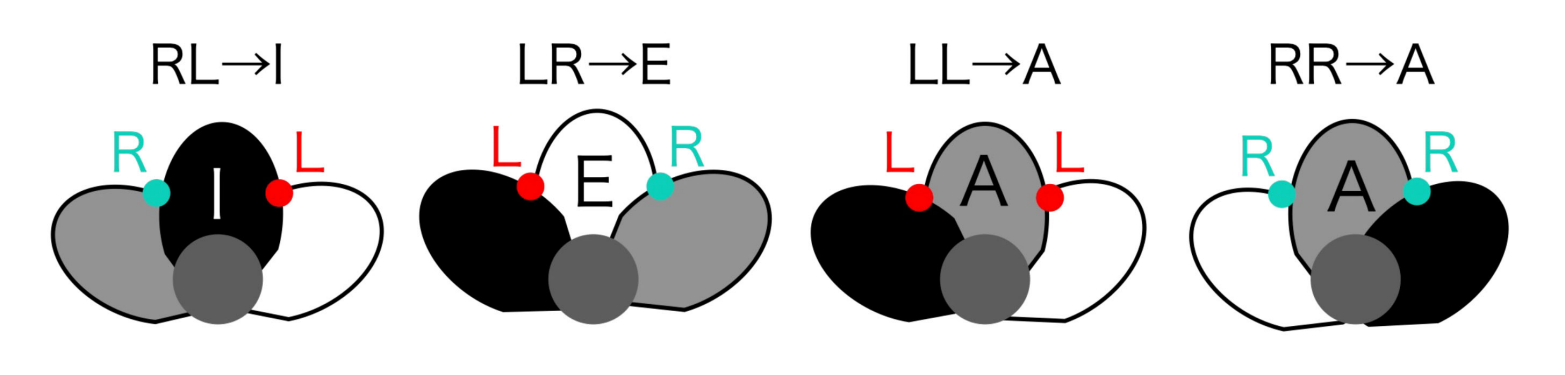
Rule for converting tepal overlapping labels to tepal labels.

Next, the class labels representing the interior-exterior relation of a pair of tepals are converted to class labels for tepals (**Figure 3**). When the circular permutation of L and R labels representing the interior-exterior relation is clockwise, a sequence of two letters, such as LR, can be converted into one of the I, E, or A labels (**Figure 11**). Following this conversion rule, the permutation represented by L and R is converted to a permutation of tepal class labels indicated by I, E, and A.

Finally, the tepal arrangement being estimated is compared with the 13 known types of tepal arrangements (**Figure 4**). Concretely, the class label permutations represented by I, E, and A are compared with the class label permutations extracted from the tepal arrangement candidates. Edit distance (Navarro, 2001) is used as the metric for the estimation. The edit distance is the minimum number of single-character edits (insertions, deletions, or substitutions) needed to change one string into another. Then, the tepal arrangement that gives the shortest edit distance is regarded as the estimation result.

As the class label permutations are circular permutations, considering the rotation and flip transformations in computing the edit distance between two permutations is necessary. Therefore, the edit distance is calculated by rotating the circular permutation. Additionally, the edit distance is calculated after a flip transformation is carried out.

## 4 Experiments

To evaluate the accuracy of the proposed method, we executed experiments using the original dataset introduced in Section 3.1. In the experiments, the accuracy of each intermediate process was evaluated. This is because the accuracy of petal arrangement estimation is heavily dependent on the accuracy of the intermediate processing steps. Next, the accuracy of the tepal arrangement estimation was evaluated after all the processing steps were combined. Lastly, the accuracy of the case was evaluated when the results for the tepal overlap positions and for the recognition of their interior-exterior relationship in the intermediate processes were manually corrected. The following sections explain each experimental condition and describe the results. The research ethics committee of the Graduate School of Informatics, Osaka Metropolitan University approved the experiments on manual correction.

### 4.1 Experimental conditions

The conditions used to evaluate the accuracy of each intermediate process are first explained. For flower detection, 200 images from the original dataset were used for training, and 3,742 images were used for testing. We used the original implementation of the YOLO v5(m) model^4^, which is pretrained on MS COCO. As the size of the input image for the YOLO v5(m) model is 256×256 pixels, the images were scaled down to 256×256 pixels using bilinear interpolation. As described in Section 3.1, the images used in the experiment were 1,000× 662-pixel landscape images. Therefore, in order not to change the aspect ratio of the images, the image was scaled down to 256 × 169 pixels. Then, black pixels were added to the scaled image to make the image size fit to the input of YOLO v5(m). In fine-tuning, following the original YOLO v5(m) implementation, blur, random size cropping, and binary histogram equalization were applied to the training data with 1% probability to augment the data. The augmentation was performed using the function included in the original implementation. Training was performed by changing the number of training epochs and batch size of the original implementation to 500 and 16, respectively. The implementation was based on the deep learning framework Pytorch 1.9.1 on Python 3.7.7. Intersection over Union (IoU), which is commonly used in object detection, was used as the evaluation metric for flower detection. When the detection result region for a given object (pixel set) is set to A, and the correct region is set to B, the IoU value is calculated using the following Equation (1):


(1) $$\text{IoU}=\frac{\left|A\cap^{\;}B\right|}{\left|A\cup^{\;}B\right|}$$

As the flower detection result is indicated by the bounding box, the IoU value was calculated using the bounding boxes of the detection result and the correct region.

Flower segmentation was evaluated on 220 images to which flower regions were manually assigned. The 220 images were different from the 200 images used for fine-tuning the flower detection method. The size of the images was 1,000×662 pixels. Segmentation was conducted by setting the flower region inside the bounding box as the foreground. We used opencv-python 4.5.3.56, which is the computer vision library OpenCV^5^ for Python, which was used for implementing segmentation. We used the function grabCut, which implements GrabCut. The initialization of grabCut, which indicates the format of the foreground settings, was set to the rectangles. The rectangles containing the flowers, which were determined manually, were given to the function. The number of algorithm iterations was set to 5. IoU was used to evaluate the accuracy, as was the case for the flower detection. As the detection result and the correct region are presented as flower regions at the pixel level, these regions were used to calculate the IoU value.

To evaluate the tepal overlap detection, 3,742 images that were manually labeled with the tepal overlap positions were used. The images used for evaluation were the same as those used for flower detection evaluation. The size of the images was 1,000 × 662 pixels. The flower segmentation method described in Section 3.3.2 was applied to the flower images, and the tepal overlap detection was performed on the segmented flower regions in the images. As for the flower segmentation, opencv-python 4.5.3.56 was used for the implementation. We used the cornerHarris function, which implements the Harris corner detector. The size of the circle radius used to determine the overlap area was 15 pixels. The F1 score (*F*_1_), which is the harmonic mean of the precision ratio and the recall ratio, was used in the accuracy evaluation. The F1 score is calculated using the following Equation (2):


(2) $$F_{1}=2\cdot \frac{\text{precision}\cdot \text{recall}}{\text{precision}+\text{recall}}$$

For the interior-exterior estimation of the tepals, 200 images were used to determine the initial parameters (meta-training), and 220 images were used for the testing (meta-testing). As the interior-exterior estimation method requires the use of images from which only flower regions are cropped, it is necessary to prepare images from which flower regions are correctly cropped for training and testing. Therefore, we used 200 and 220 manually flower-segmented images for training and testing, respectively. The 200 images used for training were the same as those used for fine-tuning the flower detection method.

Patches of size 20 × 20 pixels, whose centers were the manually labeled positions of the tepal overlap, were cropped for the meta-training and meta-testing for query sets. Note that each patch was cropped so that one side was parallel to the straight line connecting the two intersections of the circle whose center was the point of overlap and the contour, as shown in the concave image in **Figure 6**. In addition, the cropped patches were aligned so that the contour part of the tepal was on top of each cropped patch. From a single flower, 4 to 16 patches were cropped, and a total of 1,575 original patches were cropped. The synthesis patches were generated following in Section 3.3.4.1 in each flower image. The radius of the red circle shown in **Figure 8** for cropping the patches for synthesis was set to 10 and 15 pixels. Two patches of 20×20 pixels were cropped from each point of intersection of the red circle and the contour of the tepals. If the number of cropped patches was less than 50, we cropped patches around the intersections, adding perturbations, until the number of patches was increased to 50. After the cropping was done for all the overlapping points in a flower image, two types of patches were cropped, 25 patches each: contour arcs that slope upward from left to right, and contour arcs that slope upward from right to left. From each cropped patch type, one patch was selected and superimposed so that the contours of the tepals were intersected at the top to create two synthetic images: left or right in front. Synthetic patches were generated for all combinations of the cropped patches. From a single flower image, 1250 synthetic patches were generated.

For the meta-training, 200,000 tasks were prepared, and 20 tasks were prepared for the meta-testing. To construct a meta-training task, we randomly selected one image out of 200 training flower images, and then we randomly sampled 20 synthetic and 2 original patches generated from the selected flower image for the support and query sets, respectively. We iterated the above process 200,000 times, then generated 200,000 tasks for meta-training. It was acceptable for the same flower image to be selected during task generation. This is because even if the same flower image was selected, we selected the patches in such way that the combination of the patches is different from the previously generated tasks. The meta-testing tasks were generated from 220 flower images for the test. Each flower image generated a task. Each task consists of a support set of 10 synthetic patches for each class, for a total of 20 patches, and a query set of 2 original patches, one for each class.

We used the same network model as for the MAML (Finn et al., 2017). We used the implementation with Pytorch, which is a deep learning framework written in Python^6^. The code was written in Python 3, and TensorFlow v1.0+, which is a Python library for machine learning, was used to implement the model. The model consisted of 4 concatenated blocks, including batch normalization, ReLU, and 2×2 max-pooling after a 3×3 convolutional layer. As the input to the model was 84×84 pixels, we upsampled the patches from 20×20 to 84×84 pixels using bilinear interpolation. For training, no pretraining was executed, and the model was trained from scratch. The meta train iteration, meta batch size, meta learning rate, update batch size, and update learning rate were set to 200,000, 20, 0.001, 5, and 0.01, respectively. No augmentation was performed during the training. Recognition accuracy, which is the ratio of correct interior-exterior relations to the total overlap positions, was used as the evaluation metric.

We also executed an experiment that integrated all the intermediate processes and estimated the tepal arrangement in the input images. A total of 3,742 flower images were used for the evaluation; each image contained one flower. The size of the images was 1000 × 662 pixels. For each process, unless otherwise noted, the model and parameter settings were the same as for each intermediate process evaluation. Detection results, which were shown in rectangles, were given as the foreground of the flower segmentation process. After segmentation, overlapping tepals detection was performed on the segmentation results. The interior-exterior estimation was performed on the detected overlapping points. For the petal interior-exterior estimation, the same meta-training tasks that were used to evaluate the intermediate process were used, and the meta-testing tasks were generated using the 3,742 flower images, which were used for flower detection evaluation. The tepal arrangements were estimated using the results of interior-exterior estimation. In the tepal arrangement estimation, the edit distance cost of insert, delete and replace was set to 1. Recognition accuracy, which is the ratio of correct to the total number of flowers, was used as the evaluation metric.

**Figure 12:**
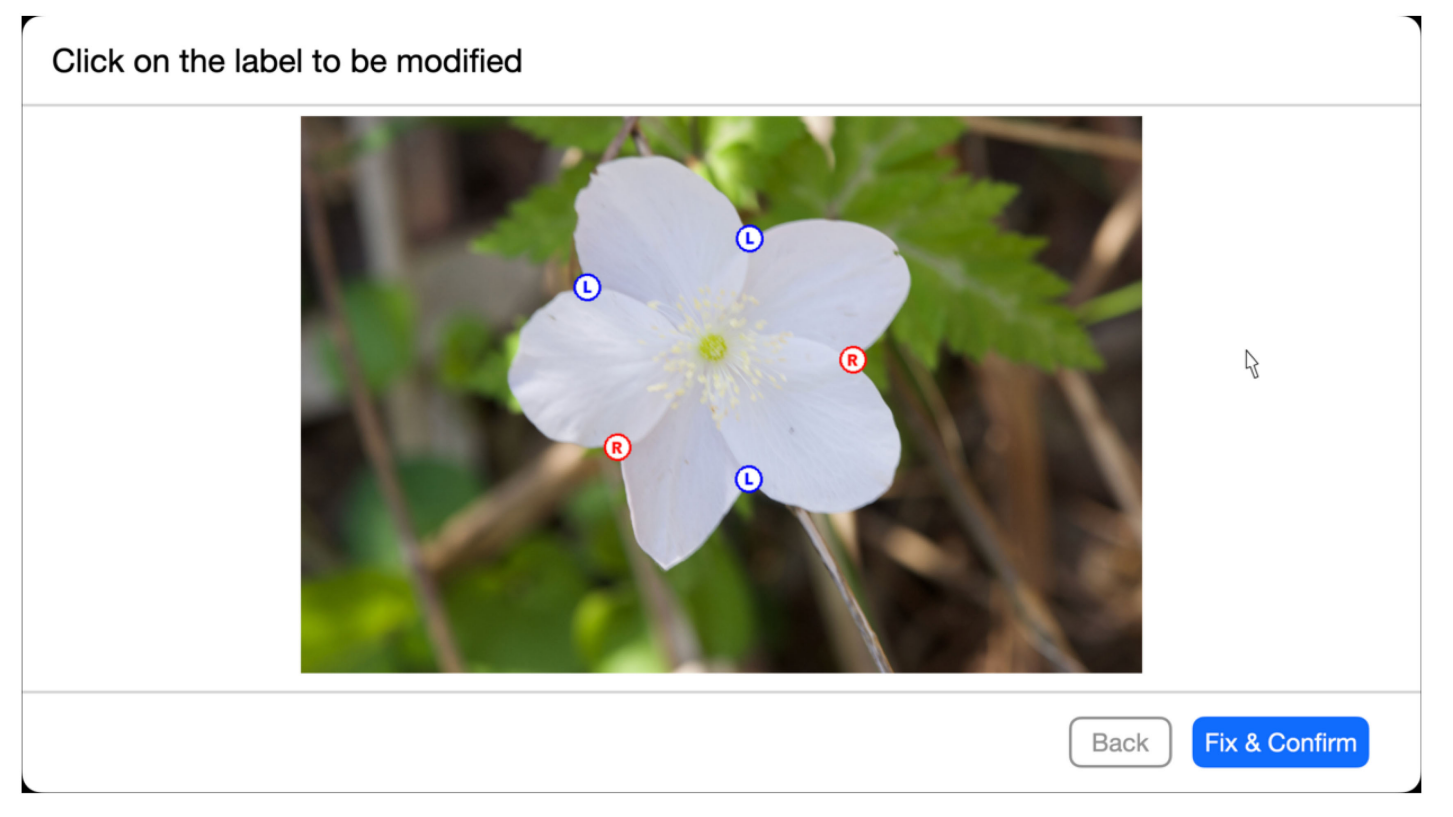
An example of the application display used in the manual correction evaluation experiment. The circles superimposed on the image indicate the detected overlap points, and their colors and letters indicate the results of the interior-exterior estimation, with the blue circled letter L and the red circled letter R on the left and on the right. If the user clicks on a circle in the estimation result, the estimation result is modified.

To evaluate manual correction, 15 of the 3,742 images were used. The size of the images was 1000 × 662 pixels. There were 9 participants in the experiment. All participants were graduate and undergraduate students majoring in computer science and were not botany experts. The experiment was conducted using a web application we developed for the experiment. **Figure 12** shows an example of what the application displays to the user in a web browser when it asks the user to modify intersections or estimation results. The web application presents an image of a flower, the position of tepal overlap detection results, and the interior-exterior estimation relationship of the petals. One can correct the detection and estimation results by mouse operation through the web application. We asked the participants to find the incorrect overlap detection results and interior-exterior relation estimation results and correct the results by mouse operation. Then, we estimate the tepal arrangements using the corrected results. The application was developed using a Python web framework Django 4.1.2 ^7^ to display images and estimation results in a browser and to collect user mouse events. The tepal arrangement estimation results from the images were computed in the same experimental environment as the integrated experiments. Recognition accuracy, which is the ratio of correctly estimated arrangements to the total test numbers of all the experiment participants, was used for the accuracy evaluation.

We used a GPU server to evaluate the deep learning-based intermediate processes: fine-tuning and evaluation of YOLO v5 for flower detection and training and evaluation of MAML for tepal interiorexterior estimation. The GPU server was equipped with NVIDIA TITIAN RTX and 24 GB of memory. We used a CPU server for the evaluation of flower segmentation, tepal overlap detection, and tepal arrangement estimation. We also used the CPU server for the integrated experiment and the web application server for the manual correction experiment. The CPU server was equipped with an Opteron 6348 and 512 GB of memory.

### 4.2 Result

**Table 1** shows the results of the accuracy evaluation of the intermediate process. The accuracy was determined to be 0.275 after all the intermediate processes were integrated, and tepal arrangement was estimated from the input images. When manual correction was applied, the accuracy was 0.711. The average time for manual correction for one flower was 48 seconds.

**Table 1:** Evaluation of intermediate process.

Intermediate process	Number of training images	Number of test images	Evaluation metric	Score
Flower detection	200	3742	IoU	0.939
Segmentation	–	220	IoU	0.974
Tepal overlap detection	–	3742	F1 score	0.870
Tepal interior-exterior relationship recognition	200	220	accuracy	0.849

## 5 Discussion

**Figure 13:**
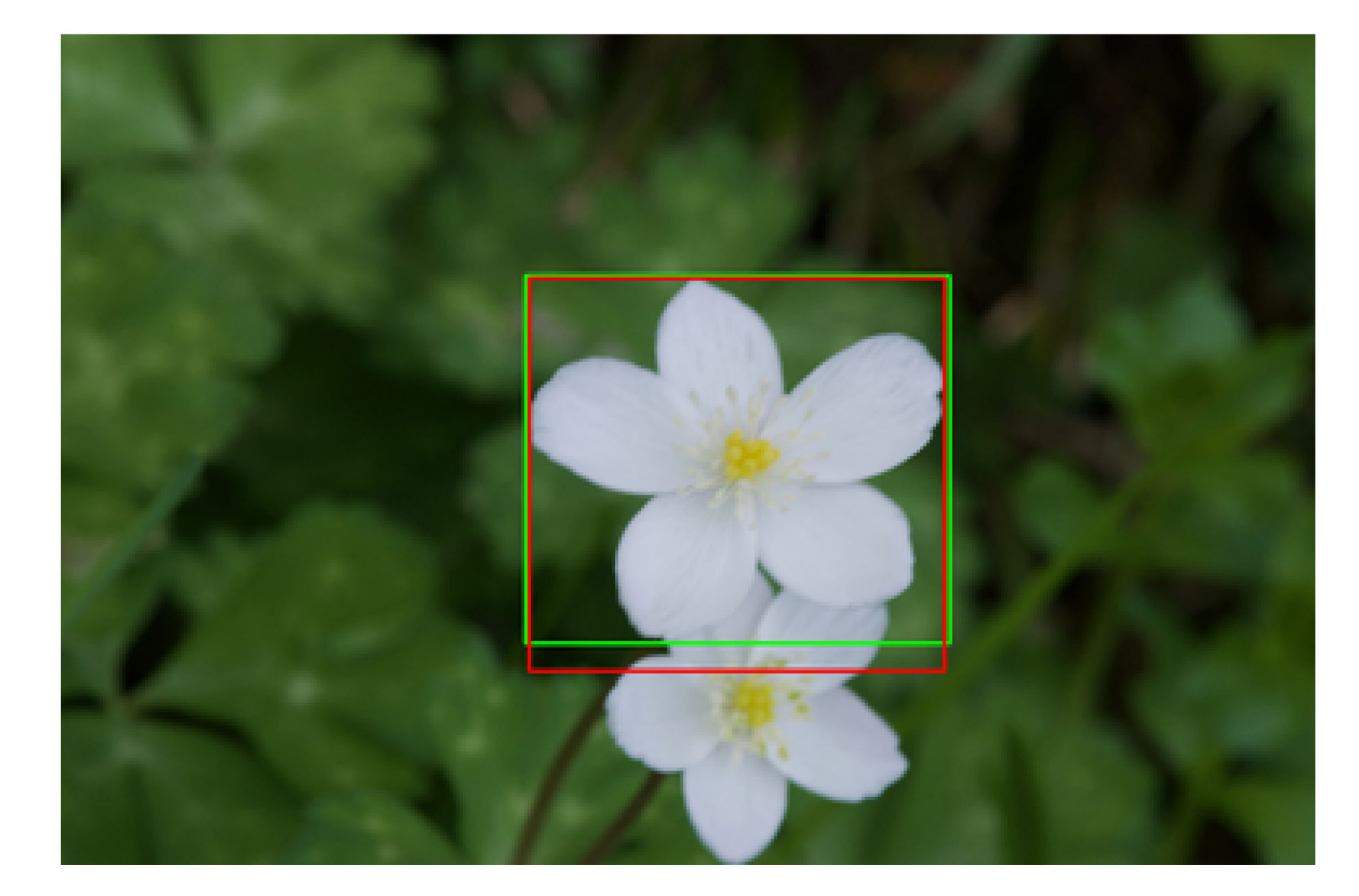
The example of the flower detection result with the lowest IoU, 0.906. The red and green rectangles show the detection result and ground truth.

**Table 1** shows that flower detection and segmentation accuracy was particularly high relative to the accuracy of each intermediate process. IoU metric is sensitive to misalignment. **Figure 13** shows an example of a flower detection result when the lowest IoU was shown, 0.906. **Figure 13** shows that the segmentation is almost perfect, as the detection result overlapped the ground truth. However, a small deviation from the ground truth decreased the IoU value. In PASCAL VOC challenge 3^8^, a common-object recognition competition, object detection was deemed to be successful when IoU was 0.5. As IoU for flower detection in this study was 0.939, the flower detection was quite accurate.

**Figure 14:**
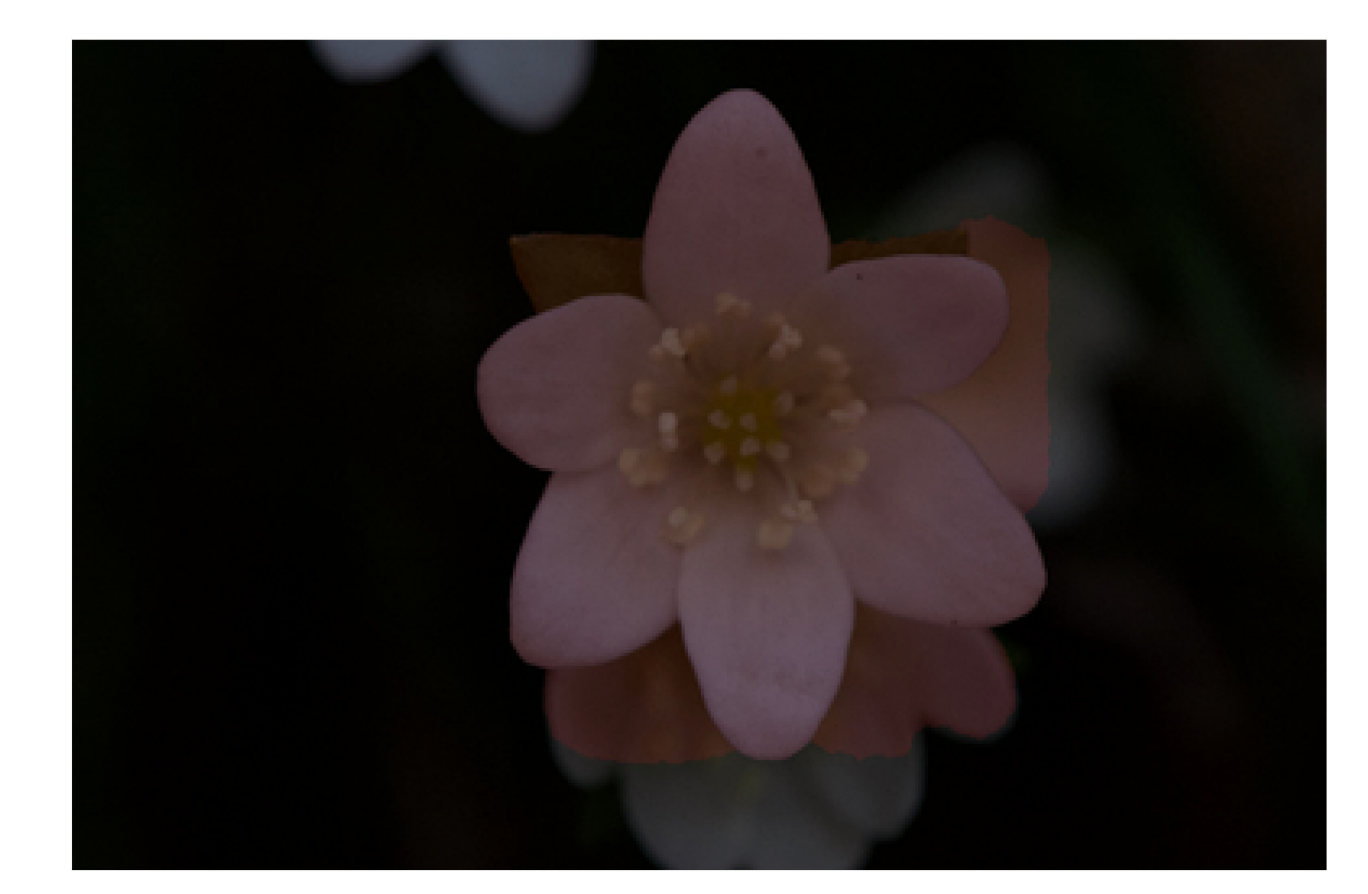
The example of the flower segmentation result with the IoU was 0.761. The light red area shows the segmented area as the foreground. The background area, which was flowers overlapping with the foreground flower, was also determined to be foreground.

As the foreground and background in the most of the flower images used for this experiment were different, as shown in **Figure 2**, flower detection was a relatively easy task. In addition, IoU value for segmentation was high at 0.974. Similar to detection, this is assumed to be because of the difference between the background and flower colors, making the segmentation task easy. However, several images failed segmentation because the background has the similar color to the foreground, as shown in **Figure 14**. GrabCut used color information to determine the foreground and background using color information.

When using Grabcut, segmentation fails if the background has a similar color to the foreground. To correctly segment such images, deep learning based segmentation methods can be effective.

**Figure 15:**
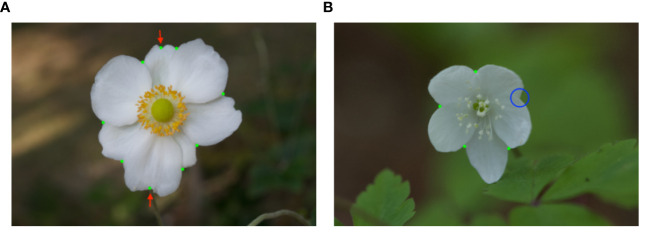
The examples of tepal overlap detection results. The green dots show the detected tepal overlap. **(A)** includes false positives, shown by the green dots with the red arrows. **(B)** includes false negative, shown by the blue circle.

**Figure 15** shows examples of the result of tepal overlap detection. This result contains errors: false positives and a false negative. In **Figure 15A**, the tepal indentations were incorrectly detected as overlaps. As the overlap detection method considers the concave points as overlaps, the overlap detection method failed on the flower images whose tepals have indentations. In **Figure 15B**, the overlap detection method missed the overlap point, shown by the blue circle. The Harris corner detector failed to detect the bluecircled corner. As the overlap detection method used in this study was a rule-based method, the detection failed when an exception occurred. To improve the accuracy, learning-based detection could be introduced.

**Figure 16:**
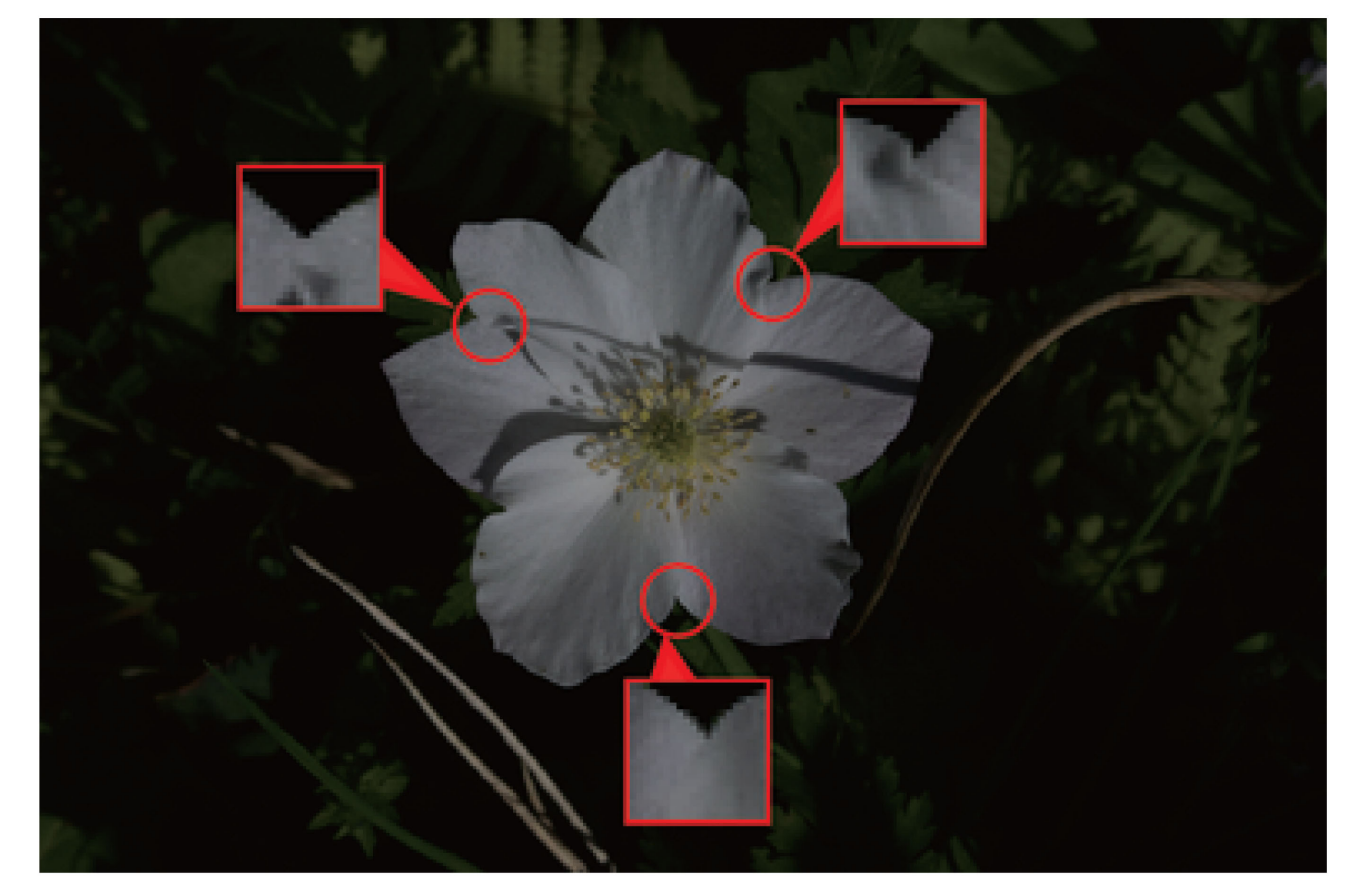
The example of failed for interior-exterior estimation. The red circles and patches show the tepal overlap points and the patches that failed to the interior-exterior estimation.

The accuracy of the interior-exterior estimation was not satisfactory enough to automate the tepal arrangement estimation. **Figure 16** shows an example with incorrect interior-exterior estimation results. In the enlarged upper two patches in **Figure 16**, the shading of the tepal folds would affect the estimation. In the lower patch, the disappearing contour of the tepal in front would affect the estimation. These changes in the appearance of the patches are caused by the lighting conditions when the images were taken. The accuracy can be improved by keeping the lighting conditions constant. However, since the flower images used in this study were taken outdoors, it is difficult to take all the images under constant lighting conditions.

In the integrated experiments, the accuracy was 0.275. This is significantly less accurate than any of the intermediate processes. To clarify the reason for the low accuracy, we theoretically calculated the accuracy when intermediate processes are integrated using the accuracy of the independent intermediate process evaluations. Since the average number of tepals in the flower images used in this study was 6.3, we consider an image of a flower with 6 tepals. The flower has 6 tepal overlapping points. Given the high accuracy of flower detection and segmentation in the experiments, we assume that they are always successful. The experimental results show that the precision and estimation accuracy for the detection of the overlapping point detection and the interior-exterior estimation are 0.889 and 0.849, respectively. For the sake of simplicity, we assume that the overlapping point detection and the interior-exterior estimation are independent. Then, the accuracy that all overlapping point detection and interior-exterior estimation will be successful for the flower is calculated by the 6 powers of the product of the detection and estimation as follows:


$${\left(0.889*0.849\right)}^{6}=\;0.18486585651.$$

The calculated accuracy is lower than the accuracy of the integrated experiment, 0.275. This is probably due to the fact that even though the overlapping point detection or the interior-exterior estimation failed, some of the flowers succeeded in the circular permutation matching of the tepal order estimation process. However, the improvement is not sufficient.

To improve the accuracy of the tepal arrangement estimation, it is essential to improve the accuracy of overlapping point detection and interior-exterior estimation. Overlap point detection could be improved by using a learning-based detection method. However, it would be difficult to improve the accuracy of interior-exterior estimation because the lighting condition when the images were taken is one of the causes of the lower accuracy of interior-exterior estimation, and the lighting condition is not constant in the outdoor environment. Therefore, improving interior-exterior relation estimation is not feasible. Another possible cause of the low accuracy is the division of the tepal arrangement estimation into overlapping point detection and interior-exterior estimation. As calculated above, each task can be assumed to be independent, so even a slight decrease in the accuracy of one task will significantly affect the accuracy when the results are integrated. A possible solution to this problem is to estimate the tepal arrangement directly from the flower image without splitting the task.

The manual correction experiment showed that manual correction could significantly improve the arrangement estimation, and the tepal arrangements can be estimated by amateurs not used to observing flowers. When the number of tepals is less than 10, as in this study, experts can estimate the tepal arrangement in a few seconds without the proposed method. Although it takes more time than with the experts, the amateurs can estimate the tepal arrangement without any flower observation experience using the proposed method with manual correction. Amateurs can estimate the tepal arrangement instead of the experts, and the experts can reduce the effort of estimating the tepal arrangement. As anyone who can estimate the tepal interior-exterior relationship can be assigned to the tepal arrangement, the estimation work can be parallelized by multiple people. Although the time for estimating the tepal arrangement by the proposed method with manual correction is longer than that of the experts, parallelization can improve the efficiency of the estimation work.

Additionally, results from manually corrected intermediate processes could be used as the ground truth data for training. If enough manually corrected data are collected, replacing the current rule-based method used for tepal interior-exterior estimation with a learning-based method would be possible. As a result, the accuracy of the estimation of tepal interior-exterior relationship would improve. In addition, as manual correction makes it easy to assign the correct tepal arrangement to the input flower image, preparing a large number of flower images with tepal arrangements would be smooth. Next, constructing an end-to-end network that performs image input and placement estimation would be possible. In the proposed method, tepal arrangement estimation was performed by combining several independent intermediate methods due to the difficulty of preparing training data to train an end-to-end network, leading to a decrease in accuracy. If an end-to-end network trained with sufficient training data and built by the proposed method with manual correction could be constructed, it would be possible to achieve highly accurate tepal arrangement estimation automatically.

## 6 Conclusion

This study focused on tepal arrangement, an indicator of the floral developmental process. Currently, specialists classify tepal arrangements in flower images manually. This requires a tremendous amount of labor. Therefore, using the image recognition method to estimate tepal arrangement from flower images was proposed to reduce specialists’ workload. As it was difficult to collect a large amount of data, a segmentation method not requiring learning and a method that could accurately recognize the interiorexterior relationships of tepals with a small amount of training data were used. Our experiment showed that, although flower detection and segmentation could be performed accurately, tepal overlap detection and the estimation of the tepal interior-exterior relationship were inaccurate, which resulted in low accuracy in the tepal arrangement estimation. However, the accuracy improved significantly when the tepal overlap detection and interior-exterior relationship recognition results were manually corrected. The results showed that manual correction can be used to assign the ground truth of a tepal arrangement to a large number of images. Thus, an end-to-end network expected to be highly accurate could be realized by using a large number of images with manually assigned tepal arrangements.

## Data availability statement

The original contributions presented in the study are publicly available. This data can be found here: https://drive.google.com/drive/u/2/folders/1IpJrhKur7POvEDyzyQl37fhSwBZvuRwZ.

## Ethics statement

The studies involving humans were approved by The research ethics committee of the Graduate School of Informatics, Osaka Metropolitan University. The studies were conducted in accordance with the local legislation and institutional requirements. The participants provided their written informed consent to participate in this study.

## Author contributions

TN: Software, Validation, Visualization, Writing – original draft. YU: Conceptualization, Funding acquisition, Investigation, Methodology, Project administration, Writing – original draft, Writing – review & editing. KF: Conceptualization, Data curation, Supervision, Writing – review & editing. MI: Conceptualization, Investigation, Methodology, Supervision, Writing – review & editing. KK: Resources, Writing – review & editing.

## Footnotes

1
https://github.com/ultralytics/yolov5.

2 3D movie is available at https://youtu.be/clYmer-nvO4.

3
https://github.com/ultralytics/yolov5.

4
https://github.com/ultralytics/yolov5.

5
https://opencv.org.

6
https://github.com/dragen1860/MAML-Pytorch.

7
https://www.djangoproject.com.

8
http://host.robots.ox.ac.uk/pascal/VOC/.
